# Circulating microRNA signatures in mice exposed to lipoteichoic acid

**DOI:** 10.1186/1423-0127-20-2

**Published:** 2013-01-04

**Authors:** Ching-Hua Hsieh, Johnson Chia-Shen Yang, Jonathan Chris Jeng, Yi-Chun Chen, Tsu-Hsiang Lu, Siou-Ling Tzeng, Yi-Chan Wu, Chia-Jung Wu, Cheng-Shyuan Rau

**Affiliations:** 1 Department of Plastic and Reconstructive Surgery, Kaohsiung Chang Gung Memorial Hospital and Chang Gung University College of Medicine, Kaohsiung City, Taiwan; 2 Business BA at University of Texas at Dallas, 800 W Campbell Road, Richardson, TX, 75080, USA; 3 Department of Neurosurgery, Kaohsiung Chang Gung Memorial Hospital and Chang Gung University College of Medicine, No.123, Ta-Pei Road, Niao-Sung District, Kaohsiung City, 833, Taiwan

**Keywords:** MicroRNAs, Lipoteichoic acid, Lipopolysaccharide, Toll-like receptor, Gram-positive bacteria, Gram-negative bacteria, Microarray

## Abstract

**Background:**

Previously, we had identified a specific whole blood–derived microRNAs (miRNAs) signature in mice following *in vivo* injection of lipopolysaccharide (LPS) originated from Gram-negative bacteria. This study was designed to profile the circulating miRNAs expression in mice exposed to lipoteichoic acid (LTA) which is a major component of the wall of Gram-positive bacteria.

**Results:**

C57BL/6 mice received intraperitoneal injections of 100 μg of LTA originated from *Bacillus subtilis*, *Streptococcus faecalis*, and *Staphylococcus aureus* were killed 6 h and the whole blood samples were obtained for miRNA expression analysis using a miRNA array (Phalanx miRNA OneArray® 1.0). Up-regulated expression of miRNA targets in the whole blood, serum and white blood cells (WBCs) of C57BL/6 and *Tlr2*^−/−^ mice upon LTA treatment in 10, 100, or 1000 ug concentrations was quantified at indicated time (2, 6, 24, and 72 h) using real-time RT-PCR and compared with that in the serum of C57BL/6 mice injected with 100 ug of LPS. A significant increase of 4 miRNAs (miR-451, miR-668, miR-1902, and miR-1904) was observed in the whole blood and the serum in a dose- and time-dependent fashion following LTA injection. Induction of miRNA occurred in the serum after 2 h and persisted for at least 6 h. No increased expression of these 4 miRNAs was found in the WBCs. Higher but not significant expression level of these 4 miRNAs were observed following LTA treatment in the serum of *Tlr2*^−/−^against that of C57BL6 mice. In contrast, LPS exposure induced moderate expression of miR-451 but not of the other 3 miRNA targets.

**Conclusions:**

We identified a specific circulating miRNA signature in mice exposed to LTA. That expression profile is different from those of mice exposed to LPS. Those circulating miRNAs induced by LTA or LPS treatment may serve as promising biomarkers for the differentiation between exposures to Gram-positive or Gram-negative bacteria.

## Background

MicroRNAs (miRNAs) are small non-coding, endogenous, single-stranded RNA molecules that regulate the activity of specific mRNA targets and play important roles in a wide range of physiologic and pathologic processes [1,2]. Alteration of miRNA expression profiles has been observed in various diseases and help distinguish between disease states [3]. Identification of these multiple miRNA changes is valuable because specific signatures of miRNA combinations unique to a normal physiological or pathological state can serve as an useful reference [4]. Recently, biochemical analyses indicate that miRNAs are resistant to RNase activity, extreme pH and temperature, extended storage, and large numbers of free-thaw cycles [5,6]. In addition, extracellular miRNAs circulate in the blood are remarkably stable [7], albeit there is presence of ribonuclease in both plasma and serum [8,9]. With the possibility to analyze multiple miRNAs in parallel to increase sensitivity and specificity by using complex miRNA expression patterns, miRNAs might constitute very useful and accessible diagnostic tools in a cluster pattern [5,10].

Early diagnosis of bacterial infection is critical for preventing further complications. Pathological processes after bacterial infection are mainly induced by structural components of bacterial cell walls [11]; In Gram-negative bacteria, these components are lipopolysaccharide (LPS) [12] and in Gram-positive flora, these components are represented by lipoteichoic acid (LTA) [13]. The appearance of LPS and LTA in the blood leads to activation of multiple intracellular signaling pathways necessary for the rapid change of the target cell functional state to provide an efficient innate immune response depends on Toll-like receptor 4 (TLR4) and Toll-like receptor 2 (TLR2), respectively. Previously, we had identified a specific whole blood–derived miRNA signature in mice exposed to LPS as there was a dose- and time-dependent upregulated expression of the miRNA targets (let-7d, miR-15b, miR-16, miR-25, miR-92a, miR-103, miR-107 and miR-451) follo-wing *in vivo* LPS injection [14]. The above-mentioned 8 miRNAs was evident as early as 2 h and persisted for at least 6 h following injection with 100 ug of LPS. In contrast, LTA exposure induced moderate expression of miR-451 but not of the other 7 miRNA targets [14].

Seeing these circulating miRNAs may serve as promising biomarkers for the differentiation between *in vivo* exposure to LPS or LTA. The present study was designed to profile the circulating miRNA expression exposure to LTA in mice.

## Methods

### Animal experiments

C57BL/6 mice were purchased from BioLasco (Taiwan). *Tlr2*^−/−^ (B6.129-Tlr2tm1Kir/J) mice were purchased from Jackson Laboratory (Bar Harbor, ME, USA). All housing conditions were established and surgical procedures, analgesia, and assessments were performed in an AAALAC-accredited, SPF facility following national and institutional guidelines. Animal protocols were approved by the IACUC of Chang Gung Memorial Hospital. LTA from different Gram-positive bacteria, including *Bacillus subtilis* (catalog no. L3265), *Streptococcus faecalis* (L4015), and *Staphylococcus aureus* (L2515) as well as LPS from different Gram-negative bacteria, including *Escherichia coli* serotype 026:B6 (L3755), *Klebsiella pneumonia* (L1519), *Salmonella enterica* serotype Enteritidis (L6761), *Serratia marcescens* (L6136), and *Pseudomonas aeruginosa* (L9143) were purchased from Sigma (St. Louis, MO, USA). When the mice gained a weight of 20–35 g and became 4–6 weeks old, they were intraperitoneally injected with 10, 100, 1000 μg of LTA reconstituted in 100 μL of phosphate-buffered saline (PBS). Animals were sacrificed at 2, 6, 24, and 72 h after LPS injection. The control group was injected with 100 μL PBS. Whole blood was drawn for miRNA expression analysis. For comparison, intraperitoneal injections of 10, 100, 1000 μg of LPS from *Escherichia coli* serotype 026:B6 (L3755) were performed in C57BL/6 mice, that were killed 6 h after injection and the whole blood was obtained for quantification of miRNA expression.

### RNA isolation and preparation

In brief, coagulated whole blood samples (1 mL per mouse) were collected at indicated times of experiment. After incubating the whole blood at 37°C for 1 h and centrifugating at 3,000 rpm for 10 min, the white blood cells (WBCs) were slowly removed from the corresponding layers and the serum was extracted and stored at −80°C before use. Total RNA was extracted from whole blood, serum and WBCs by using the RNeasy Mini kit (Qiagen, Hilden, Germany). Purified RNA was quantified by measuring the absorbance at 260 nm by using an SSP-3000 Nanodrop spectrophotometer (Infinigen Biotechnology, Inc., City of Industry, CA, USA). For miRNA array analyses, the quality of purified RNA was assessed using a Bioanalyzer 2100 (Agilent Technologies, Santa Clara, CA, USA). Total RNA (2 μg) was reverse transcribed into cDNA by using the TaqMan miRNA Reverse Transcription Kit (Applied Biosystems, Foster City, CA, USA). Target miRNA was reverse transcribed using sequence-specific stem-loop primers. miRNA cDNA (10 ng) for each target was used for real-time PCR.

### miRNA microarray analysis

Mouse genome-wide miRNA microarray analysis was performed by Phalanx Biotech with the Mouse & Rat miRNA OneArray® 1.0 (Phalanx Biotech Group, Hsinchu, Taiwan) contains a total of 2,319 probes, including 135 experimental control probes and 728 unique miRNA probes from mouse and 348 from rat (miRBase Release 12.0). Briefly, fluorescent targets were prepared from 2.5-μg total RNA samples by using the miRNA ULS™ Labeling Kit (Kreatech Diagnostics, Amsterdam, Netherlands). Labeled miRNA targets enriched using NanoSep 100K (Pall Corporation, Port Washington, NY, USA) were hybridized to the microarray with Phalanx hybridization buffer by using the OneArray® Hybridization Chamber. After overnight hybridization at 37°C, non-specific binding targets were by 3 washing steps (Wash I: 37°C, 5 min; Wash II: 37°C, 5 min and 25°C, 5 min; and Wash III: rinse 20 times). The slides were dried by centrifugation and scanned using Axon 4000B scanner (Molecular Devices, Sunnyvale, CA, USA). The Cy5 fluorescent intensities of each spot were analyzed using GenePix 4.1 software (Molecular Devices). The signal intensity of each spot was processed using the R program. We filtered out spots for which the flag was <0. Spots that passed the criteria were normalized using the 75% scaling normalization method. Normalized spot intensities were converted into gene expression log_2_ ratios for the control and treatment groups. Spots with log_2_ ratios ≥ 1 or log_2_ ratio ≤ −1 and *P*-value < 0.05 are analyzed further. These differentially expressed miRNAs were subjected to hierarchical cluster analysis using average linkage and Pearson correlation as a measure of similarity. The GEO accession number for the microarray data is GSE41837.

### Quantification of miRNA expression

The miRNA expression of the whole blood, serum and WBCs was quantified by real-time RT-PCR using Applied Biosystems 7500 Real-Time PCR System (Applied Biosystems) to verify the miRNA targets with up-regulated expression that were detected through the miRNA array from whole blood following injection of LTA different doses (10, 100, and 1000 μg) or at indicated survival times (2, 6, 24, and 72 h). Expression of the miRNAs in serum following intraperitoneal injections of LPS at doses of 10, 100, and 1000 μg were measured for comparison. Expression of each miRNA of the whole blood samples or WBCs was represented relative to the expression of U6 small nuclear RNA (*U6 snRNA*) as an internal control. For the serum, 25 fmol of single stranded cel-miR-39 synthesized by Invitrogen (Carlsbad, CA 92008) were spiked into 400 μL of serum an internal control for the expression of each miRNA. We calculated the fold-expression of induction as the relative expression value obtained from 6 samples in comparison with that from the control group. Intergroup group comparisons were performed using analysis of variance (ANOVA) and an appropriate post hoc test to compensate for multiple comparisons (SigmaStat; Jandel, San Rafael, CA, USA). *P*-values < 0.05 were considered significant.

## Results

### Up-regulated miRNA targets in microarray analysis

**Figure 1:**
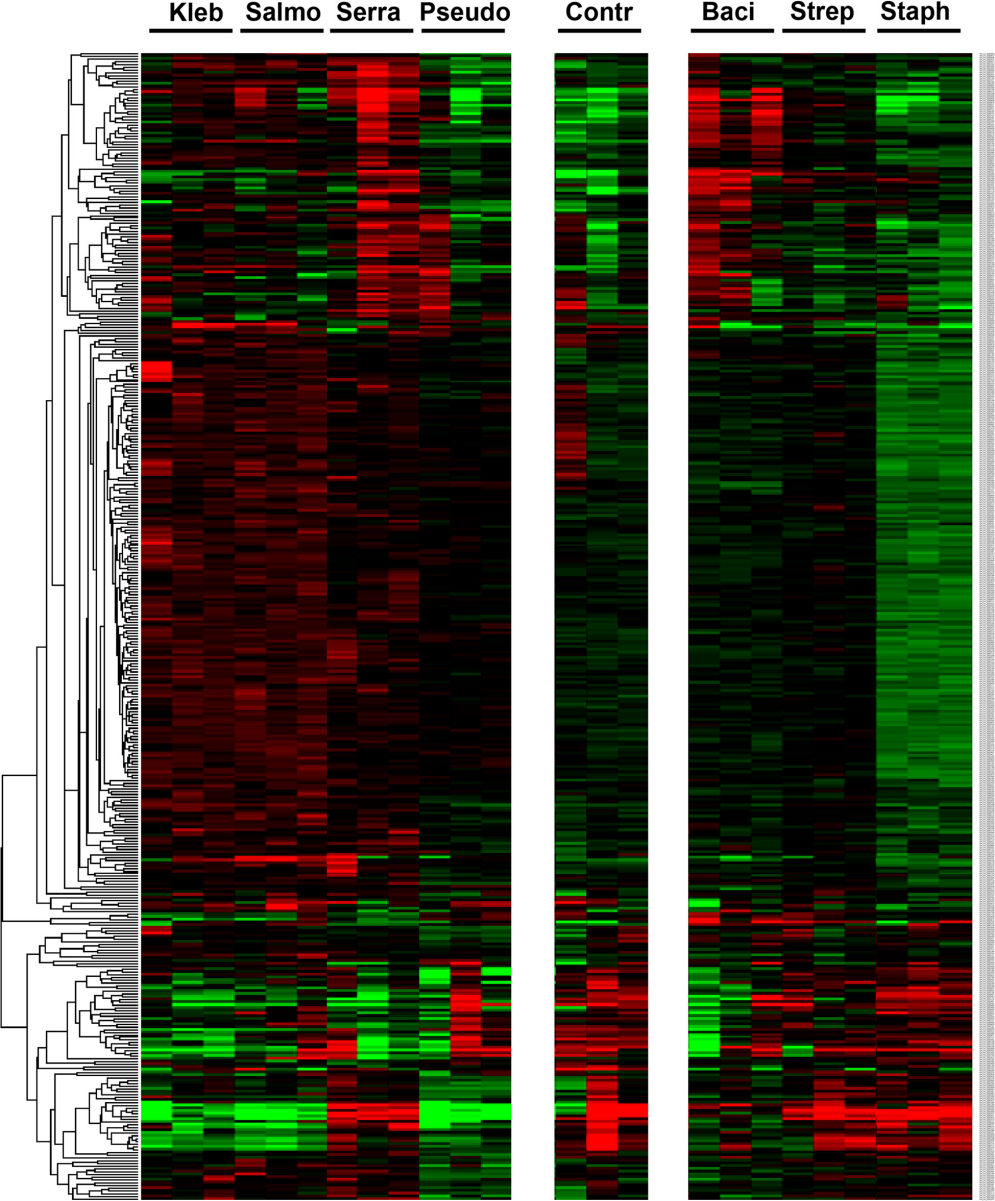
**Hierarchical cluster analysis of significant expression of miRNA in whole blood of C567BL/6 mice 6 h after injection of 100-μg LPS or LTA, originated from different Gram-negative and Gram-positive bacteria, respectively.** The indicated miRNA name by the corresponding gene probe in Phalanx array could be found in: http://www.phalanx.com.tw/Products/MRmiOA_Probe.php. Kleb: *Klebsiella pneumonia*; Salmo: *Salmonella enterica* serotype Enteritidis; Serra: *Serratia marcescens*; Pseudo: *Pseudomonas aeruginosa*; Bacl: *Bacillus subtilis*; Strep: *Streptococcus faecalis*; Staph: *Staphylococcus aureus*; Contr: sham control with PBS injection.

Expression of miRNA was considered significantly different when values for all samples from the whole blood of experimental mice at 6 h after 100 μg LTA or LPS injections were more than double of those for the controls (n=3 for each subgroup). The hierarchical cluster analysis of all significant miRNAs is shown in Figure 1, which illustrates miRNAs differentially expressed in the whole blood after LTA or after LPS injection. Unsupervised hierarchy clustering was used to separate the LTA- or LPS-treated samples into different groups. LTA from *Bacillus subtilis* (L3265) demonstrated a different expression to those from *Streptococcus faecalis* (L4015) and *Staphylococcus aureus* (L2515) in the unsupervised hierarchy clustering (Figure 1). The up-regulated miRNA targets more than double of those of the control is shown in Table 1. There were 4 miRNAs (miR-451, miR-668, miR-1902, and miR-1904) showed significantly increased expression in the whole blood of mice exposed to LTA originated from different Gram-positive bacteria.

**Table 1:** **Up-regulated miRNA targets more than double of those from the controls in microarray analysis of the whole blood of experimental mice at 6 h after the injection of 100 μg of LTA from *Bacillus subtilis*, *Streptococcus faecalis*, and *Staphylococcus aureus *(n=3 for each subgroup)**

**LTA induced miRNA targets**	***Bacillus subtilis***	***Streptococcus faecalis***	***Staphylococcus aureus***
miR-451	1.22	1.54	1.25
miR-668	1.52	1.06	1.14
miR-1902	1.37	1.44	1.05
miR-1904	1.27	1.61	1.01

The value is expressed as fold (log_2_) of the miRNA targets expression than those in the control.

### Expression profiles of miRNAs

**Figure 2:**
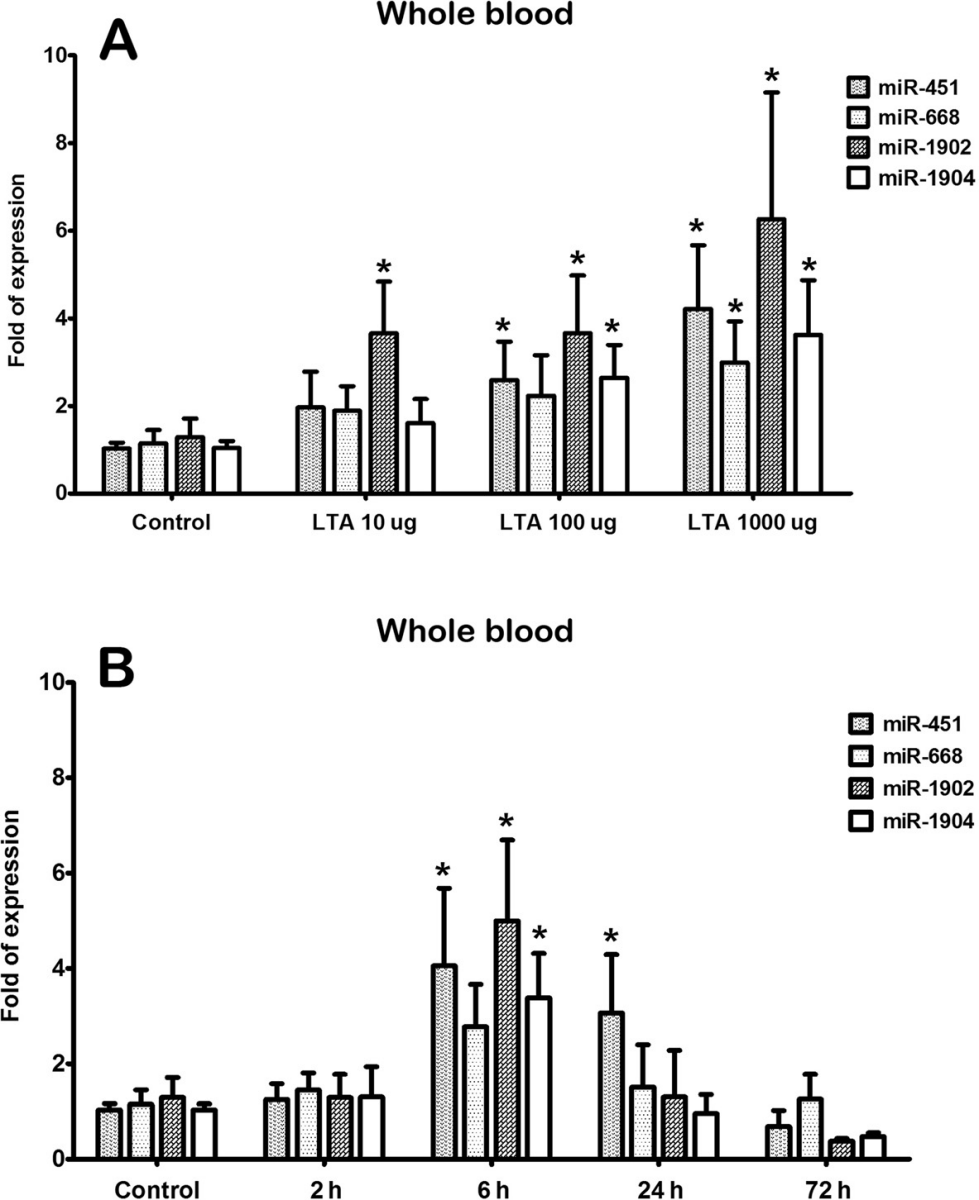
**(A) Dose- and (B) time-dependent upregulation of miRNA expression in the whole blood.** Using real-time RT-PCR, the expression of the up-regulated miR-451, miR-668, miR-1902 and miR-1904 identified using miRNA microarray was detected in the whole blood at 6 h following injection of 10, 100, 1000 μg of LTA; mice were killed at the indicated survival times (2, 6, 24, and 72 h) following 100 μg of LTA injection. Bars represent means ± SEM of 6 experiments; *, *P* < 0.05 vs. control.

Expression of miRNAs in whole blood, serum and WBCs following LTA injection was quantified using real-time RT-PCR to verify selected up-regulated miRNA targets with at least 2-fold increase in expression (miR-451, miR-668, miR-1902, and miR-1904). In the whole blood, following injection with 10 μg of LTA, there was significantly increased expression in miR-1902, but not in miR-451, miR-668 or miR-1904 (Figure 2A). Upon 100 ug of LTA injection, significantly increased expression around 2- to 3-fold was observed in miR-451, miR-1902, and miR-1904, but not in miR-668 (Figure 2A). When exposed to 1000 ug of LTA, all these four miRNAs (miR-451, miR-668, miR-1902, and miR-1904) had a significant expression around 3- to 6-fold than the control (Figure 2A). Induction of miR-451, miR-1902, and miR-19042, but not miR-668, was evident at 6 h following injection with 100 ug of LTA (Figure 2B). At 24 h, only miR-451 continued to be significantly expressed in the whole blood (Figure 2B). No up-regulation of these 4 miRNA targets was detected at 2 h as well as 72 h after LTA injection (Figure 2B).

**Figure 3:**
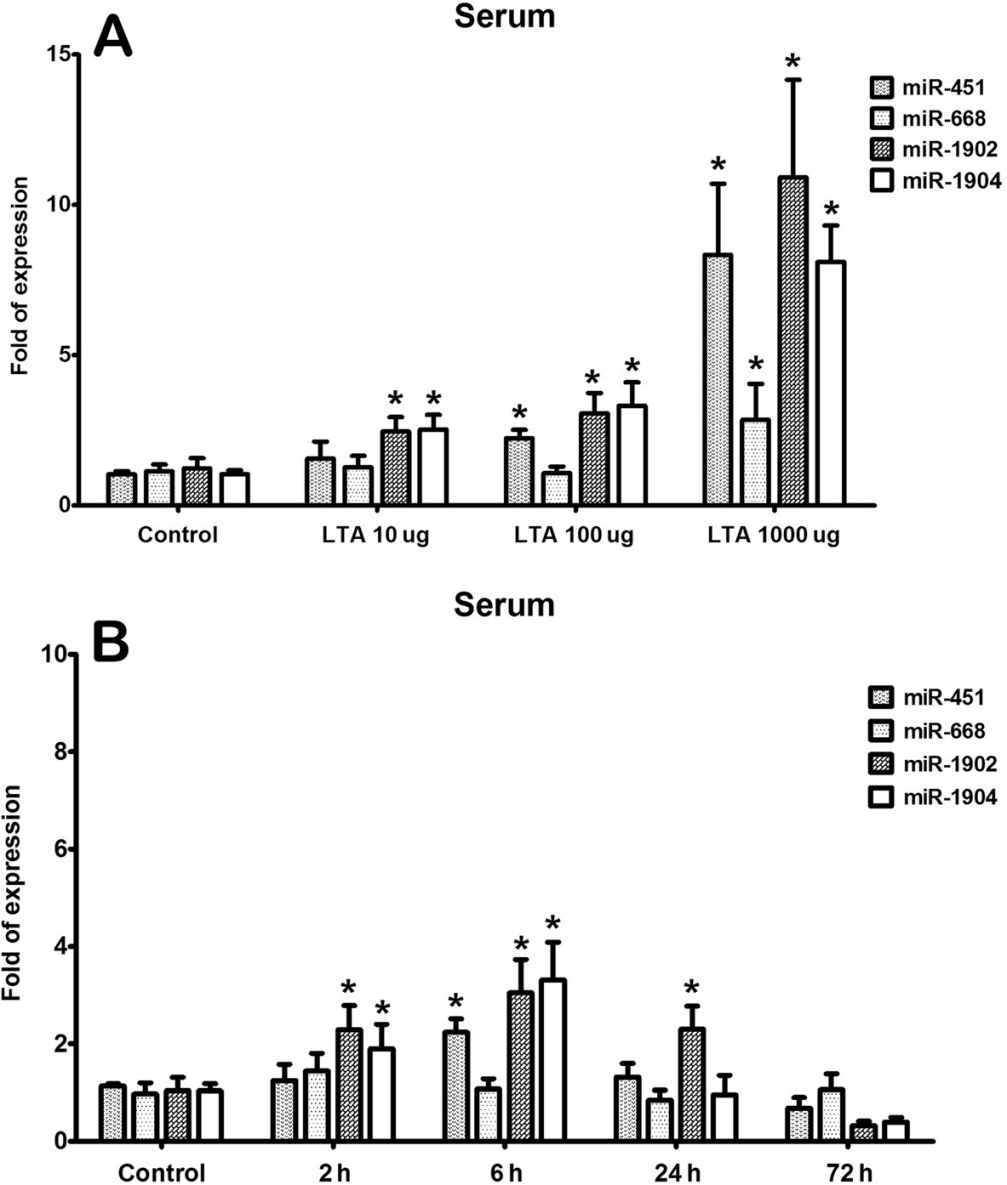
**(A) Dose- and (B) time-dependent upregulation of miRNA expression in the serum.** Using real-time RT-PCR, the expression of the up-regulated miR-451, miR-668, miR-1902 and miR-1904 was detected in the serum at 6 h following injection of 10, 100, 1000 μg of LTA; mice were killed at the indicated survival times (2, 6, 24, and 72 h) following 100 μg of LTA injection. Bars represent means ± SEM of 6 experiments; *, *P* < 0.05 vs. control.

In the serum, the dose- and time-dependent pattern of expression of these 4 miRNAs following LTA injection is similar to those in the whole blood, except that significantly increased expression of miR-1904 was found following injection with 10 μg of LTA (Figure 3A) and induction of miR-1902 and miR-19042 was evident as early as at 2 h following injection with 100 ug of LTA (Figure 3B). In addition, at 24 h, only miR-1902, but not miR-451 which was observed in the whole blood, continued to be significantly expressed in the serum (Figure 3B). No up-regulation of these 4 miRNA targets was detected in the serum at 72 h after LTA injection (Figure 3B).

### miRNA expression in the serum and WBCs in C57BL/6 and Tlr2 knockout mice

**Figure 4:**
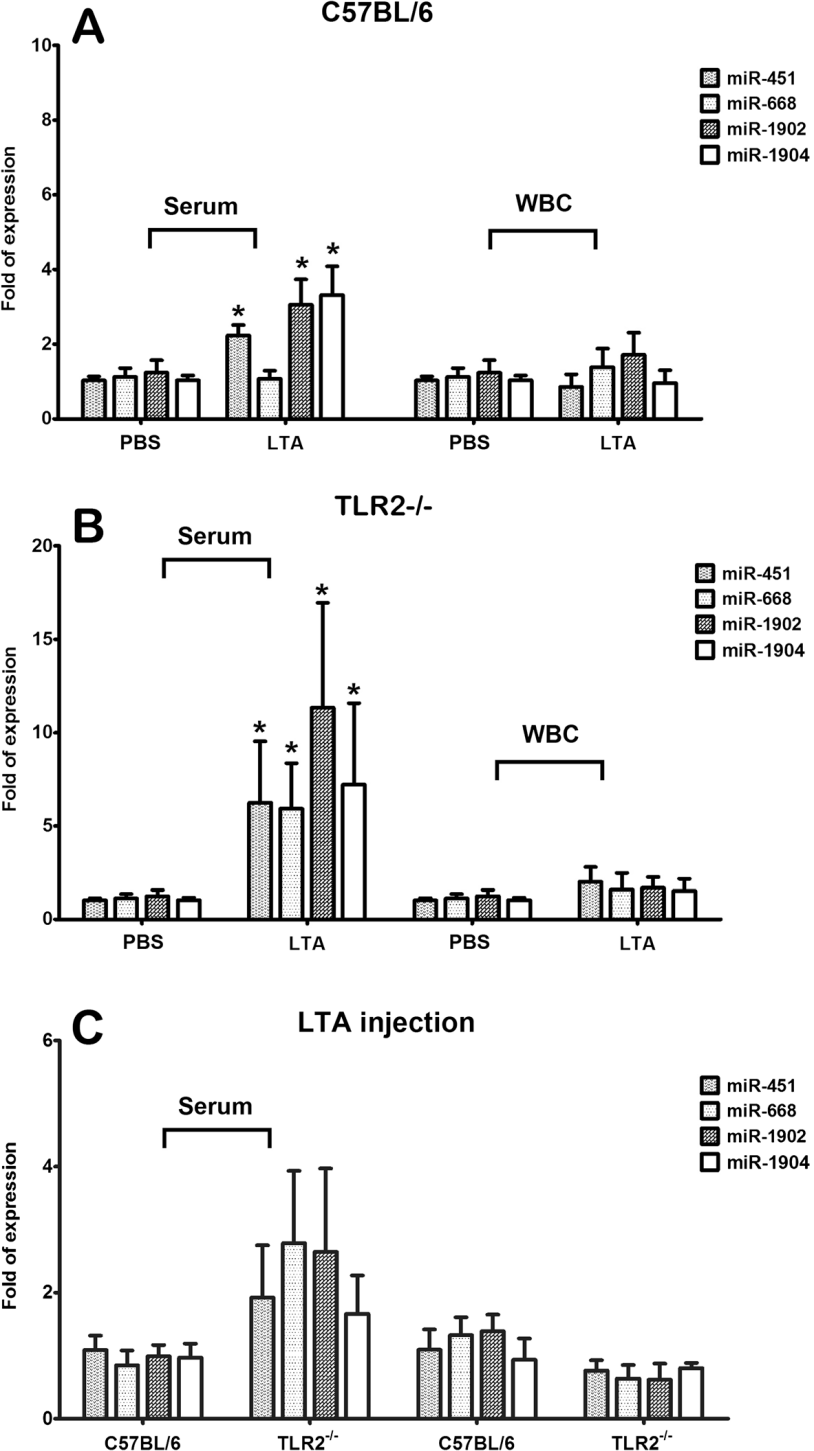
**Expression of miR-451, miR-668, miR-1902, and miR-1904 of serum and WBCs from C57BL/6 and *Tlr2 ***^**−/− **^mice from real-time RT-PCR experiments 6 h after exposure to 100 μg of LTA or phosphate-buffered saline (PBS); **, *P*< 0.01 vs. control of C57BL/6 or *Tlr2*^**−/−**^**.** (**A**) C57BL/6 mice receiving injection of PBS or LTA; (**B**) *Tlr2*^−/−^ mice receiving injection of PBS or LTA; (**C**) C57BL/6 or *Tlr2*^−/−^ mice upon injection of LTA.

In contrast to the expression of miR-451, miR-1902, miR-1904, but not miR-668, in serum of C57BL/6 at 6 h after exposure to 100 μg of LTA, no significantly up-regulation of these 4 miRNA targets was detected in the WBCs after LTA injection, when compared with those in C57BL/6 receiving PBS injection (Figure 4A). In *Tlr2*^−/−^ mice, significantly higher expression of miR-451, miR-668, miR-1902, and miR-1904 in the serum were observed following LTA injection with around 6- to 10-fold of expression against that from the *Tlr2*^−/−^ mice injected with PBS (Figure 4B). Notably, higher expression of these 4 miRNA targets in the serum of *Tlr2*^−/−^ mice was noted after LTA injection than those in the serum of C57BL/6 mice, albeit not statistically significant (Figure 4C). No significant change of these 4 miRNA targets was detected in the WBCs of *Tlr2*^−/−^ against C57BL/6 mice following LTA injection (Figure 4C).

### miRNA expression after LPS injection

**Figure 5:**
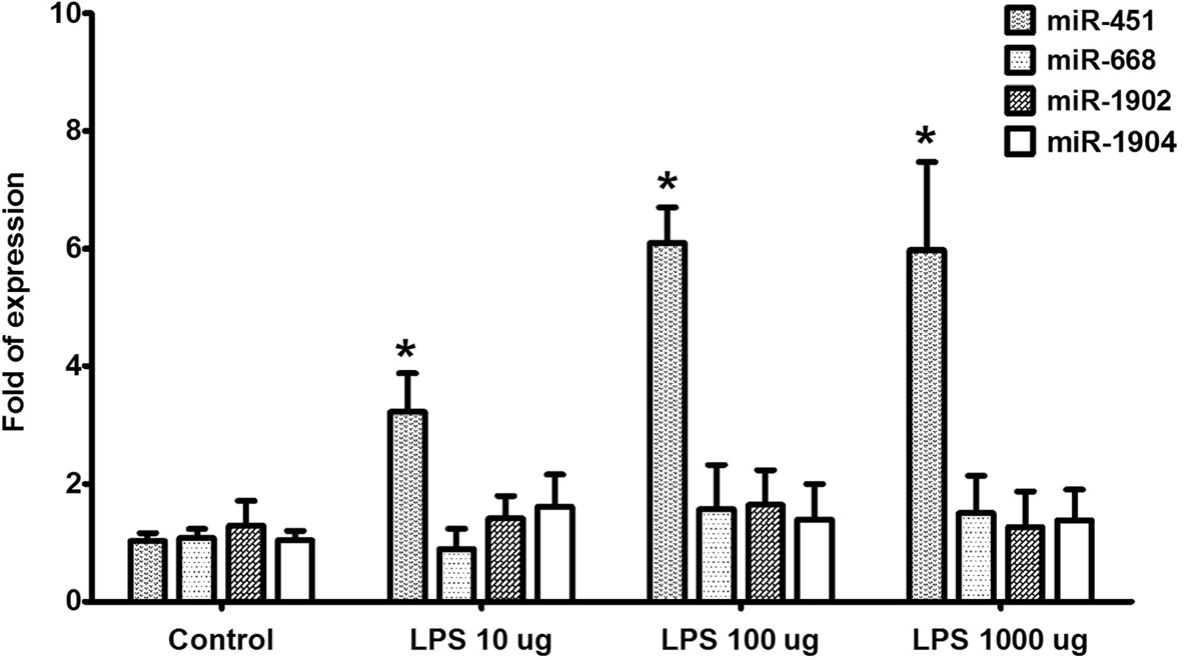
Expression of miR-451, miR-668, miR-1902, and miR-1904 of serum from C57BL/6 mice using real-time RT-PCR experiments 6 h after exposure to 10, 100, and 1000 μg LPS from *Escherichia coli *serotype 026:B6; *, *P *< 0.05 vs. control.

To investigate that whether LPS originating from Gram-negative bacteria *Escherichia coli* serotype 026:B6 (L3755) induces expression of miR-451, miR-668, miR-1902, and miR-1904, the serum was obtained for real-time PCR at 6 h following intraperitoneal injections of 10, 100, or 1000 μg LPS. The results showed that LPS significantly induced miR-451 expression at concentrations of 10, 100 and 1000 μg but not up-regulated the expression of the other 3 miRNA targets (Figure 5).

## Discussion

While LPS and LTA induce similar inflammatory responses, the signaling and sensing of LPS and LTA differ significantly. In previous study, we had demonstrated that expression of multiple miRNAs (let-7d, miR-15b, miR-16, miR-25, miR-92a, miR-103, miR-107, and miR-451) is significantly altered in the whole blood of mice after exposure to LPS in a dose- and time-dependent fashion [14]. Besides, LTA did not up-regulate the expression of these miRNA targets, except miR-451, in the concentration up to 1000 ug [14]. In this study, we demonstrated that expression of miR-451, miR-668, miR-1902, and miR-1904 is significantly altered in the whole blood and serum of mice after exposure to LTA in a dose- and time-dependent fashion. Additionally, LPS significantly induced miR-451 expression at concentrations of 10, 100 and 1000 μg but not increase the expression of the other 3 miRNA targets. Therefore, a specific signature of miRNAs in the circulation of mice exposed to LPS and LTA may serve as promising as biomarkers for their exposure.

Among these 4 miRNA targets, miR-451 had been reported to be a promising biomarker for microRNAs involved in lung tissue infected with pathogen as *Actinobacillus pleuropneumoniae*[15]. miR-451 had also been found to direct a negative regulatory cascade to tune the cytokine production of dendritic cells including IL-6, TNF, CCL5/RANTES, and CCL3/MIP1α [16]. However, miR-451 also presents with a significant level in RBCs and comprised the major source of variation in its level measured in the circulation and deterred its use as a circulating biomarker [17]. Up-regulation miR-668 in the peripheral blood of mice exposed to mainstream smoking had been found to reflect the dynamic pathological changes in smoking-related interstitial fibrosis [18], but so far there was no linkage of miR-668 to any infectious disease had been reported in the literature. Notably, miR-451 was both induced by LPS and LTA treatment in this study and the expression of miR-668 was significantly induced in the whole blood and in the serum by the high dose of LTA in 1000 ug concentration, but not by lower to medium dose of LTA in 10 and 100 ug concentrations. Therefore, the use of miR-451 or miR-668 as a biomarker to differentiate the exposure to LPS or LTA is not suitable. In addition, expression signature of miR-1902 and miR-1904 may be useful in differentiating infections caused by gram-positive bacteria to those by gram-negative bacteria. In the mouse jejunum infections with *Eimeria papillata*, miR-1902 expression was able to serve as one of those biomarkers to reflect the garlic treatment of this illness [19]. A study on mouse-adapted avian influenza H9N2 with the mutation as substitution of E627K in the PB2 protein confirmed the E627K to be responsible for the higher virulence of H5N1 in mice. The mutation PB2-E627K located on the microRNAs binding site responsible for the altered recognition of mmu-mir-1904 may explain why the mutation of PB2-E627K play the key role in virulence to mice [20]. In this study, the increased miR-1902 and miR-1904 of the whole blood could only be found 6 h after LTA treatment; however, using spiked-in cel-miR-39 as an internal control, significant expression of miR-1902 and miR-1904 in the serum may be detectable as early as 2 h following exposure to LTA. We believed this phenomenon is due to the disturbance of measurement by the cellular component of the whole blood as well as the use of *U6 snRNA* as an internal control, since these up-regulated miRNAs are located in the serum but not in the WBCs and the *U6 snRNA* is not a good candidate as a normalization control for miRNAs in the serum [21,22].

In our previous work, in TLR4 receptor knockout mice, five of eight miRNAs (i.e. let-7d, miR-25, miR-92a, miR-103, and miR-107) was significantly lower following exposure to LPS, with unchanged levels of the other three miRNAs [14]. However, in this study, higher expression of miR-451, miR-668, miR-1902, and miR-1904 in the serum were observed in *Tlr2*^−/−^ against C57BL/6 mice following LTA injection. This observation indicated that these 4 miRNAs were not induced directly by the TLR2 signaling. Because most pathogens can engage multiple TLRs, some authors speculate that those miRNAs induced by TLR singalling may regulate the strength, location, and timing of TLR responses and avoid excess pro-inflammtory repsonses as a negative feedback loop by targeting the TLR signaling molecules and shutting down several TLR pathways [23]. In addition, different expressed miRNAs may work together to control the expression of TLR signal components [23]. Whether the higher expression of miR-451, miR-668, miR-1902, and miR-1904 in the serum upon LTA stimulation is due to lack of the repressed targets of negative feedback loop in *Tlr2*^−/−^ mice is speculated but lack of evidence so far and required further investigation and validation.

When compared to those upon LPS treatment in our previous work [14], the circulating miRNA signature after LTA treatment in this study has some additional limitations. First, LPS is a stronger stimulator than LTA to induce the expression of circulating miRNAs, considering the up-regulated 8 miRNAs of the whole blood showed approximately 5- to 12-fold increase in expression 6 h after 100 and 1000 μg LPS injection, these four miRNAs (miR-451, miR-668, miR-1902, and miR-1904) had a less prominent 2- to 6-fold increase upon LTA treatment. LTA has a simpler structure that typically consists of a polyglycerolphosphate (PGP) chain that is linked via a glycolipid anchor to the bacterial membrane [24]. There was a small difference between the chemical structures of PGP-type LTA from different Gram-positive bacteria. For example, additional N-acetylglucosamine modifications of the hydroxyl groups at position C2 of PGP chain are found in *B*. *subtilis* but not in the *S*. *aureus*[25]. Although LTA is a major immunostimulating component in the cell wall of Gram-positive bacteria, it is expressed on not only pathogenic but also nonpathogenic Gram-positive bacteria. LTAs from *Staphylococcus aureus* (pathogenic), *Bacillus subtilis* (non-pathogenic), or *Lactobacillus plantarum* (beneficial) carry differential potencies in the stimulation of TLR2 and expression of inflammatory cytokines [26] as well as nitric oxide signaling pathway [27]. In this study, LTA from *Bacillus subtilis* demonstrated a different expression to those from *Streptococcus faecalis* and *Staphylococcus aureus*. However, with a weaker stimulator for miRNA expression, the sensitivity and specificity of complex miRNA expression in diagnosis may be limited. Second, unlike TLR4 comprised by the homogenous dimers, TLR2 is involved in the recognition of a wide range of pathogen-associated molecular patterns (PAMPs) derived from Gram-positive bacteria beside LTA, including bacterial lipoproteins [28,29], and peptidoglycan [30], albeit the latter for TLR2 signaling is still an issue of debate [31]. This high versatility of ligand recognition by TLR2 is possibly due to the ability of TLR2 to form heterodimers with TLR1 or TLR6 [32]. Third, LTAs are commonly referred to as membrane-associated polymers characteristic of gram-positive bacteria, just as lipopolysaccharides are believed to be ubiquitous among gram-negative bacteria [24]. However, the number of gram-positive bacteria now known to lack classical LTA is steadily increasing [24,33], that may limit the use of circulating miRNAs as the biomarkers for exposure to Gram-positive bacteria.

Despite accumulating evidence of miRNAs in the circulation, the origin, the composition, and the function of these circulating extracellular miRNAs remains poorly understood [34]. Although the expression of circulating miRNAs is thought to reflect extrusion of miRNAs from relevant remote tissues or organs or disease processes [6], currently, little is known regarding the biologic roles of these molecules at distant sites in the body [35]. Three sources of circulating miRNAs had been suggested, including (1) passive leakage from broken cells due to tissue injury, chronic inflammation, cell apoptosis or necrosis, or from cells with a short half-life, such as platelets; (2) active secretion via microvesicles, including exosomes and shedding vesicles; (3) active secretion using a microvesicle-free, RNA-binding protein-dependent pathway, where a significant portion of circulating miRNAs in plasma is associated with high-density lipoprotein (HDL) [36], Argonaute2 (AGO2) [37,38], and another RNA-binding protein, nucleophosmin 1 (NPM1) [39]. From this study and our previous work, although we had identified different expression signature of the circulating miRNAs following LPS and LTA exposure as promising biomarkers at the very first step; however, further investigation is required to understand the location of expressed miRNAs in the circulation and to clarify their origins and physiological roles.

## Conclusions

A specific circulating miRNA signature was identified in mice exposed to LTA. That expression profile is different from those of mice exposed to LPS. Those circulating miRNAs induced by LTA or LPS treatment may serve as promising biomarkers for the differentiation between exposures to Gram-positive or Gram-negative bacteria.

## Competing interests

The authors declare no potential conflict of interests.

## Authors’ contributions

CHH was responsible for the writing of the manuscript. YCC participated in the analysis and interpretation of the data. JCJ and THL participated in the animal surgery and acquisition of the study specimens. YCC and CJW participated in the real-time RT-PCR experiment. YCW and SLT were involved in the acquisition of the miRNA array and whole genome expression data. CSR contributed to the design and coordination of the data acquisition and analysis. All authors read and approved the final manuscript.
